# MMTV-Wnt1 and -ΔN89β-Catenin Induce Canonical Signaling in Distinct Progenitors and Differentially Activate Hedgehog Signaling within Mammary Tumors

**DOI:** 10.1371/journal.pone.0004537

**Published:** 2009-02-19

**Authors:** Brigitte Teissedre, Alicia Pinderhughes, Angela Incassati, Sarah J. Hatsell, Minoti Hiremath, Pamela Cowin

**Affiliations:** Departments of Cell Biology and Dermatology, New York University School of Medicine, New York, New York, United States of America; Health Canada, Canada

## Abstract

Canonical Wnt/β-catenin signaling regulates stem/progenitor cells and, when perturbed, induces many human cancers. A significant proportion of human breast cancer is associated with loss of secreted Wnt antagonists and mice expressing MMTV-Wnt1 and MMTV-ΔN89β-catenin develop mammary adenocarcinomas. Many studies have assumed these mouse models of breast cancer to be equivalent. Here we show that MMTV-Wnt1 and MMTV-ΔN89β-catenin transgenes induce tumors with different phenotypes. Using *axin2/conductin* reporter genes we show that MMTV-Wnt1 and MMTV-ΔN89β-catenin activate canonical Wnt signaling within distinct cell-types. ΔN89β-catenin activated signaling within a luminal subpopulation scattered along ducts that exhibited a K18^+^ER^−^PR^−^CD24^high^CD49f^low^ profile and progenitor properties. In contrast, MMTV-Wnt1 induced canonical signaling in K14^+^ basal cells with CD24/CD49f profiles characteristic of two distinct stem/progenitor cell-types. MMTV-Wnt1 produced additional profound effects on multiple cell-types that correlated with focal activation of the Hedgehog pathway. We document that large melanocytic nevi are a hitherto unreported hallmark of early hyperplastic Wnt1 glands. These nevi formed along the primary mammary ducts and were associated with Hedgehog pathway activity within a subset of melanocytes and surrounding stroma. Hh pathway activity also occurred within tumor-associated stromal and K14^+^/p63^+^ subpopulations in a manner correlated with Wnt1 tumor onset. These data show MMTV-Wnt1 and MMTV-ΔN89β-catenin induce canonical signaling in distinct progenitors and that Hedgehog pathway activation is linked to melanocytic nevi and mammary tumor onset arising from excess Wnt1 ligand. They further suggest that Hedgehog pathway activation maybe a critical component and useful indicator of breast tumors arising from unopposed Wnt1 ligand.

## Introduction

Wnts are a family of secreted proteins that regulate tissue patterning and homeostasis. The canonical Wnt pathway operates by inhibiting proteolysis of cytoplasmic β-catenin, which enters the nucleus and regulates transcription through Lef/Tcf DNA binding partners. It is well documented that canonical Wnt/β-catenin signaling is required for the viability of particular stem cells, and forced activation of this pathway can expand stem/progenitors, alter cell fate and induce tumorigenesis [1]–[3].

Multiple lines of evidence demonstrate roles for Wnt/β-catenin signaling in mammary development and breast cancer [4], [5]. Multiple *Wnts* are expressed throughout mammary development. Mice expressing Wnt inhibitors, or deficient in Lef-1, show defective embryonic mammary development, and loss of the Wnt coreceptor, LRP5/6, impairs postnatal development [6]–[9]. Both loss- and gain-of-function studies have established roles for Wnt4 and Wnt5a in ductal side branching and for β-catenin signaling in alveologenesis and survival [10]–[15]. Although β-catenin mutations have not been found in breast cancer, pathway activation due to loss of the extracellular Wnt antagonist, sFRP1, is a frequent event [4], [5], [16]. In mice, expression of *Wnt1* and stabilized β-catenin (ΔN89β-catenin) under the control of the mouse mammary tumor virus LTR (MMTV) induces precocious mammary development and adenocarcinoma formation [13], [17], [18]. Tumors induced by both transgenes are enriched in side-population content and cells expressing primitive cell markers that exhibit greater colony-forming capabilities. Collectively, these studies have lead to the hypothesis that canonical Wnt/β-catenin signaling predisposes mice to breast cancer by amplifying stem/progenitor populations [4], [18]–[22].

Recent studies have shown that only a minor subpopulation of human breast tumor cells can propagate tumors [23]. Such tumor-initiating cells (TICs) share with normal stem cells the ability to self-renew and to generate differentiated progeny. Although commonly referred to as cancer stem cells (CSCs), it is unclear whether TICs derive from stem cells and/or from less potent progeny that acquire stem cell properties during transformation. Moreover, the contribution of distinct cancer stem cells to breast cancer heterogeneity remains obscure. Candidate mammary stem and progenitor populations have been identified by ultrastructural features, expression of stem cell antigen-1 (Sca-1), and ability to efflux Hoechst 33342 dyes, which upon cell sorting generates a side-population [24]–[26]. Recent sorting studies of murine mammary cells have identified a subpopulation, with a lineage-depleted (Lin^−^)/CD24^low^/CD29/49f^high^/ Sca-1^−^/keratin (K)14^+^ profile, that are enriched in “mammary repopulating units” (MRUs), which have a parent-progeny relationship with a second Lin^−^/CD24^high^/CD29/49f^low^ subpopulation of alveolar-limited progenitors expressing a predominantly luminal K18^+^ profile [21], [27]. Other studies have described luminal progenitor populations with CD24^high^/CD133^−^/K18^+^ and Lin^−^/CD24^+^/CD29^low/^CD61^+^ K14^+^ profiles [28], [29]. A study on human breast strongly supports the concept of a multipotent stem cell located within a ductal luminal niche that expresses multiple keratins [30].

Recently the connection between the physiological role of Wnt signaling in stem cells and its capacity to induce cancer when upregulated has been exploited to identify intestinal stem cells by virtue of their expression of a Wnt-responsive gene, Lgr5, that was initially found to be upregulated in colonic tumors [31]. This study suggests that identifying cell-types that respond to Wnt signaling may be an alternative route to identify and illuminate the relationship between mammary stem/progenitor cells and cancer stem cells. With this aim in mind, we employed Wnt-responsive conductin/Axin2 reporter genes to identify and isolate cells showing transcriptional response to expression of MMTV-ΔN89β-catenin and MMTV-Wnt1 transgenes. Our results show that these transgenes lead to activation of the canonical Wnt pathway in distinct cell-types displaying progenitor and stem cell characteristics and induce tumors with different phenotypes. A further source of disparity between these tumors arises from the unique ability of Wnt1 to influence multiple cells types within the gland. We show that these profound effects correlate with focal induction of Hedgehog (Hh) pathway activity within subpopulations of stromal and basal (K14^+^/p63^+^) cells found exclusively within the Wnt-1 tumor microenvironment, as well as, in and around melanocytic hyperplastic nevi, which form a hallmark of all MMTV-Wnt1 mammary glands. These data show that Hh pathway activation is linked to Wnt-1-induced mammary tumor onset and nevi formation and suggest that Hedgehog pathway activation maybe a critical component and useful marker of breast tumors arising from unopposed Wnt1 ligand.

## Results

### MMTV-Wnt1 and MMTV-ΔN89β-catenin show phenotypic disparity

**Figure 1:**
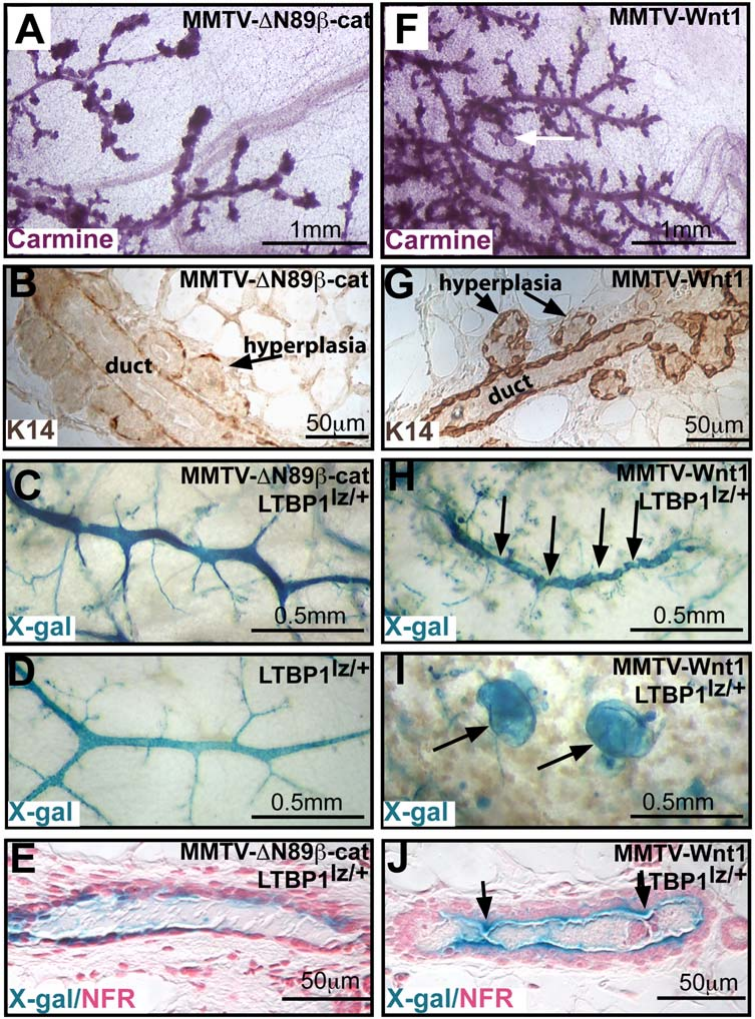
Phenotypic differences between MMTV-ΔN89β-catenin and MMTV-Wnt1 hyperplasias. Mammary glands from 12 week-old virgin MMTV-ΔN89β-catenin (MMTV-ΔN89β-cat) (A–E) and MMTV-Wnt1 (F–J) mice. Mammary whole mounts stained with carmine (A, F) or X-Gal (C, D, H, I). Sections stained immunohistochemically with anti-K14 antibodies to detect the basal cells (B, G) or with X-Gal to detect luminal LTBP1-lacZ expression and nuclear fast red (E, J). Note mammary glands from MMTV-ΔN89β-catenin mice show alveolar hyperplasia (A, B) but their ductal system (C, E) is comparable to controls (D). Mammary glands from MMTV-Wnt1 mice show hyperbranching (F), K14^+^ cell ensheathment (G), ductal constriction (arrows H, J) and large ductal cysts (arrows F, I).

**Figure 2:**
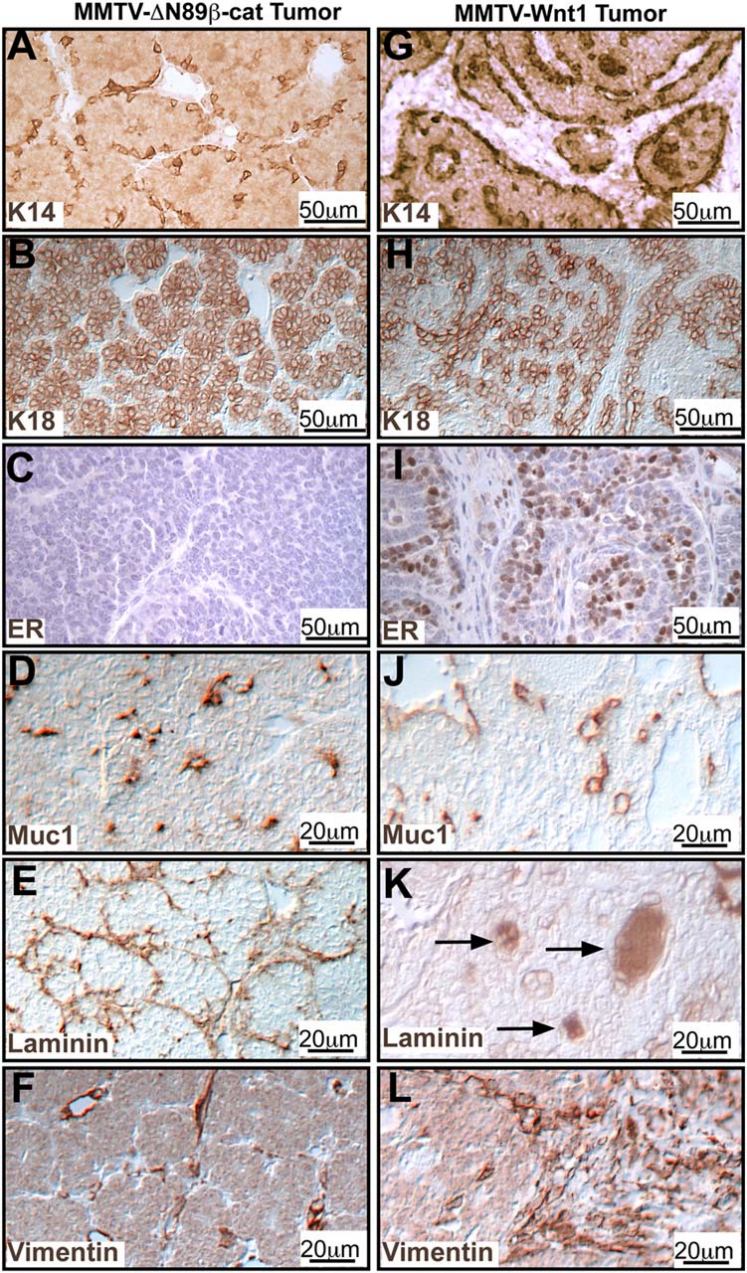
MMTV-ΔN89β-catenin and MMTV-Wnt1 tumors have distinct cellular composition. Tumor sections from MMTV-ΔN89β-catenin (MMTV-ΔN89β-cat) (A–F) and MMTV-Wnt1 (G–L) stained with antibodies as indicated against K14, K18, estrogen receptor (ER), MUC1, laminin, and vimentin. Note distorted laminin deposition (Arrows in K) and stromal hyperplasia (L).

MMTV-Wnt1 and MMTV-ΔN89β-catenin induce precocious mammary development and adenocarcinoma formation [13], [17]. However, the two types of transgenic lines differ in their lactational competence [13], [17]. This functional disparity led us to examine the respective mammary phenotypes for morphological differences that may yield insight into the corresponding pathobiological mechanisms. In glands expressing MMTV-ΔN89β-catenin acini budded directly from ductal borders (Fig. 1A), and were associated with spindle-shaped K14^+^ basal cells (Fig. 1B), which resembled flattened myoepithelial cells of normal alveoli. However, the ducts, visualized by a ductal luminal cell marker, latent transforming growth factor-β-binding protein (LTBP)1-lacZ (Fig. 1C, E), were similar to those of controls (Fig. 1D). In contrast, MMTV-Wnt1 expression induced pronounced ductal changes, including hyperbranching (Fig. 1F). These hyperplasias were encased in a coherent thick K14^+^ cell layer (Fig. 1G), which resembled the myoepithelial layers ensheathing normal ducts. Ductal contortion (Fig. 1H), cyst formation (Fig. 1I), and lumenal occlusions (Fig. 1J) were prominent. The cellular composition and architecture of the tumors induced by the transgenes also showed subtle but important differences. Tumors from MMTV-ΔN89β-catenin mice contained many more K18^+^ than K14^+^ cells and were hormone receptor-negative (Fig. 2A–C). These frequently retained well-differentiated acinar polarization, with luminal cells expressing apical mucin (MUC)1 (Fig. 2D) and showing basal deposition of laminin V (Fig. 2E). Stromal cells, detected by vimentin antibodies, were sparse (Fig. 2F). Tumors from MMTV-Wnt1 mice displayed bi-layered papillary arrays of cells, showed an equal ratio of K14^+^/K18^+^ cells and expressed hormone receptors (Fig. 2G–I). They showed loss of polarized organization; indicated by reduced MUC1 expression (Fig. 2J), frequent laminin V accumulation in patches (Fig. 2K), and stromal hypertrophy (Fig. 2L). In conclusion, expression of MMTV-ΔN89β-catenin induced expansion of hormone-receptor-negative, K18^+^ cells, which maintained alveolar features and were lactationally competent, whereas MMTV-Wnt1 expression significantly expanded K14^+^ cells in addition to both hormone-receptor-positive and -negative K18^+^ cells and lead to perturbed ductal formation and lactational failure.

### MMTV-Wnt1 and MMTV-ΔN89β-catenin activate the canonical signaling pathway within different cell-types

**Figure 3:**
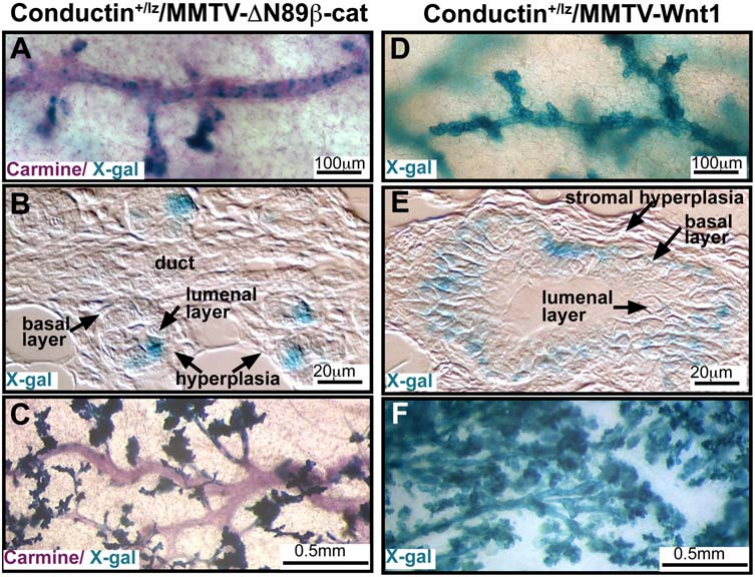
Conductin-lacZ is expressed in K18^+^ subpopulations in MMTV-ΔN89β-catenin and K14^+^ subpopulations in MMTV-Wnt1 hyperplasia. Canonical signaling is activated in disparate cell-types. Sections of hyperplastic glands from (A–C) Conductin^+/lz^/MMTV-ΔN89β-catenin mice, and (D–F) Conductin^+/lz^/MMTV-Wnt1 mice stained with X-gal to detect Conductin-lacZ expression (blue), a reporter of canonical Wnt signaling, and stained with antibodies against K14 and K18 (brown) as indicated. Note the stromal hypertrophy surrounding Wnt1 hyperplastic ducts (E).

**Figure 4:**
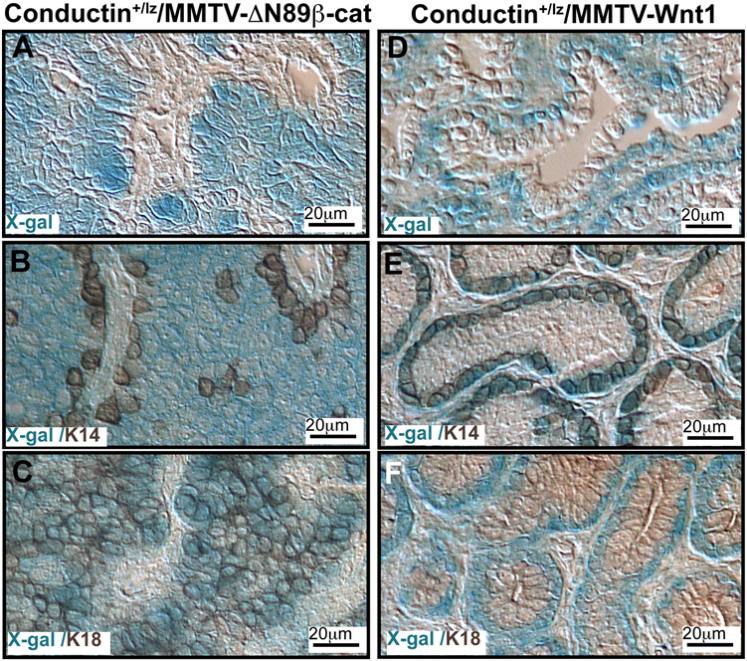
Conductin-lacZ is expressed in K18^+^ subpopulations in MMTV-ΔN89β-catenin and K14^+^ subpopulations in MMTV-Wnt1 tumors. Sections of tumors from (A–C) Conductin^+/lacZ^/MMTV-ΔN89β-catenin, and (D–F) Conductin^+/lacZ^/MMTV-Wnt1 mice stained with X-gal to detect Conductin-lacZ expression (blue), a reporter of canonical Wnt signaling, and stained immunohistochemically with antibodies against K14 and K18 (brown) as indicated.

Based on the disparate phenotypes described above, we hypothesized that ΔN89β-catenin and Wnt1 transform different progenitors. The MMTV-LTR is expressed within mammary luminal epithelium. Therefore β-catenin's transcriptional effects are restricted to cells within this layer, whereas Wnt1, a secreted factor, can act in an autocrine and/or paracrine fashion in any cells that expresses a Wnt1 receptor. To determine which cell-types respond to the expression of these transgenes via activation of the canonical pathway, we crossed MMTV-ΔN89β-catenin and MMTV-Wnt1 mice to Axin2-d2EGFP transgenic and *Conductin^+/lz^* heterozygous *lacZ* knock-in reporter lines. The *Axin2/Conductin* gene is expressed constitutively in response to canonical Wnt/β-catenin signaling and its product negatively regulates the pathway [32], [33]. Despite uniform expression of MMTV-ΔN89β-catenin within the luminal epithelium [13], transcriptional response, detected by *Conductin-lacZ* reporter expression, was restricted to a subpopulation of luminal cells scattered along secondary and tertiary ducts (Fig. 3A). In early hyperplasia, approximately one luminal cell per alveolus expressed *Conductin-lacZ* (Fig. 3B) and these cells became more prominent as the hyperplasia increased but the main ducts remained devoid of β-catenin signaling (Fig. 3C). In contrast, mammary glands from MMTV-Wnt1 mice showed a strikingly uniform *Conductin-lacZ* expression pattern along the entire mammary ductal system (Fig. 3D and F) and the reporter was found exclusively within basal cells (Fig. 3E). These distinct cellular patterns of transcriptional response were maintained in tumors (Fig. 4A–F). In tumors from MMTV-ΔN89β-catenin mice, *Conductin-lacZ* was expressed within cells expressing the luminal marker K18 and absent from K14^+^ cells (Fig. 4A–C). Wnt1 tumors showed the converse expression pattern; *Conductin-lacZ* expressing cells were restricted to a single K14^+^/K18^−^ layer of the bilayered papillary tumors (Fig. 4D–F). In conclusion, these data show that a subset of K18^+^ cells respond to ΔN89β-catenin. K18^+^ luminal epithelial cells from MMTV-Wnt1 mice fail to respond, suggesting they lack a Wnt1 receptor, or that autocrine canonical pathway activity is suppressed by non-canonical antagonism within this cell-type. In contrast, K14^+^ cells show a robust paracrine canonical response to Wnt1.

### MMTV-ΔN89β-catenin and MMTV-Wnt1 amplify cells with distinct CD24/49f stem/progenitor profiles

**Figure 5:**
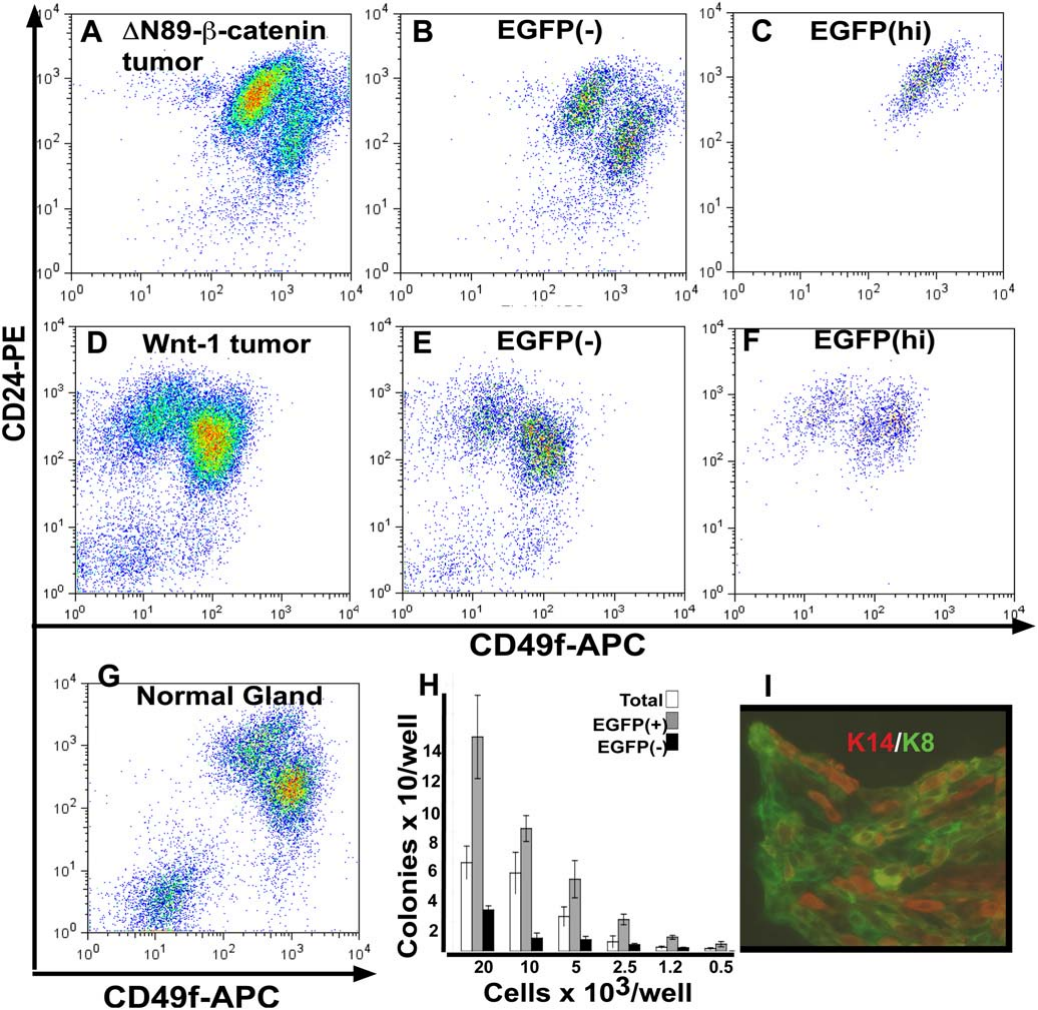
MMTV-ΔN89β-catenin and MMTV-Wnt1 tumors show expansion of distinct CD24/CD49f cell populations. CD24/CD49f marker profile of (A) total, (B) EGFP- and (C) EGFP^hi^ cells from MMTV-ΔN89β-catenin tumors. Note the enrichment and upward shift of EGFP^hi^ cells within the CD24^high^/CD49f^low^ subpopulation. CD24/CD49f marker profile of (D) total, (E) EGFP- and (F) EGFP^hi^ cells from MMTV-Wnt1 tumors. Note EGFP^hi^ cells are shifted rightward within the CD24^high^/CD49f^low^ subpopulation and upward within the CD24^low^/CD49f^high^ subpopulation. CD24/CD49f marker profile of (G) total cells from a *wt* littermate. (H) EGFP^+^ cells from MMTV-ΔN89β-catenin tumors show higher colony forming efficiency than EGFP^(−)^ and total sorted cells. (I) Immunofluorescence analysis shows these EGFP^+^ cells are generate colonies containing K14^+^ and K8^+^ cells.

To further compare the cell-type composition of tumors, we isolated mammary epithelial cells (MECs) from tumors of MMTV-ΔN89β-catenin and MMTV-Wnt1 mice and investigated their expression of CD24 and CD49f. These markers separate MECs from *wt* mice into two roughly equal populations by flow cytometry: CD24^high^CD49f^low^ and CD24^low^CD49f^high^ (Fig. 5G). The most rightward and upward shifted cells within each of these populations have been reported to be enriched in colony-forming cell (CFC) comprising alveolar progenitors and multipotent mammary repopulating units (MRUs), respectively [27]. ΔN89β-catenin tumors were enriched for CD24^high^CD49f^low^ MECs (Fig. 5A) (62% compared to 37% CD24^low^CD49f^high^). In contrast, Wnt1 tumors were enriched for CD24^low^CD49f^high^ MECs (Fig. 5D) (65%, compared to 35% CD24^high^CD49f^low^). To characterize cells undergoing an active transcriptional response to expression of these transgenes, we crossed MMTV-Wnt1 and MMTV-ΔN89β-catenin lines to the Axin2-d2EGFP reporter mouse, isolated tumor MECs from the bitransgenic progeny and analyzed them for enhanced green fluorescent protein (EGFP) and CD24/CD49f expression by flow cytometry. In contrast to the even distribution of EGFP^−^ cells from MMTV-ΔN89β-catenin mice between the two major CD24/CD49f cell populations (Fig. 5B), cells expressing high levels of EGFP^hi^ (top 15%) (Fig. 5C) fell exclusively within the CD24^high^CD49f^low^ subpopulation and showed an upward rightward shift in mean fluorescence intensity, suggestive of CFCs. To test the functional significance of these observations, we compared the ability of EGFP^+^, EGFP^−^ and total MECs from MMTV-ΔN89β-catenin mice to form colonies at limiting dilution. EGFP^+^ cells showed 3-5-fold greater colony forming efficiency compared to total sorted cells, and a corresponding depletion of colony-forming efficiency was found within the EGFP^−^ cell population (Fig. 5H). Immunofluorescence analysis showed that EGFP^+^ cells produced colonies composed of mixed progeny, with some cells expressing K8 and others K14 (Fig. 5I). We conclude that ΔN89β-catenin signaling cells within tumors show marker expression profiles that mirror those of CFC alveolar progenitors from normal gland and are bipotent, giving rise to luminal and basal cell types *in vitro*. In contrast, EGFP^−^ (Fig 5E) and EGFP^hi^ (Fig. 5F) cells from MMTV-Wnt1 mice were distributed in both CD24/CD49f subpopulations. However, the EGFP^hi^ (Fig. 5F) cells were skewed to the right side of the CD24^high^CD49f^low^ population and those within the CD24^low^CD49f^high^ subpopulation showed an upward rightward shift. These shifts are consistent with the profiles of CD61^+^/K14^+^ progenitors and of MRUs that have been shown in previous reports to have CSC capabilities [22], [27], [28], [34].

### MMTV-Wnt1 expression induces Hh pathway activation within the tumor

**Figure 6:**
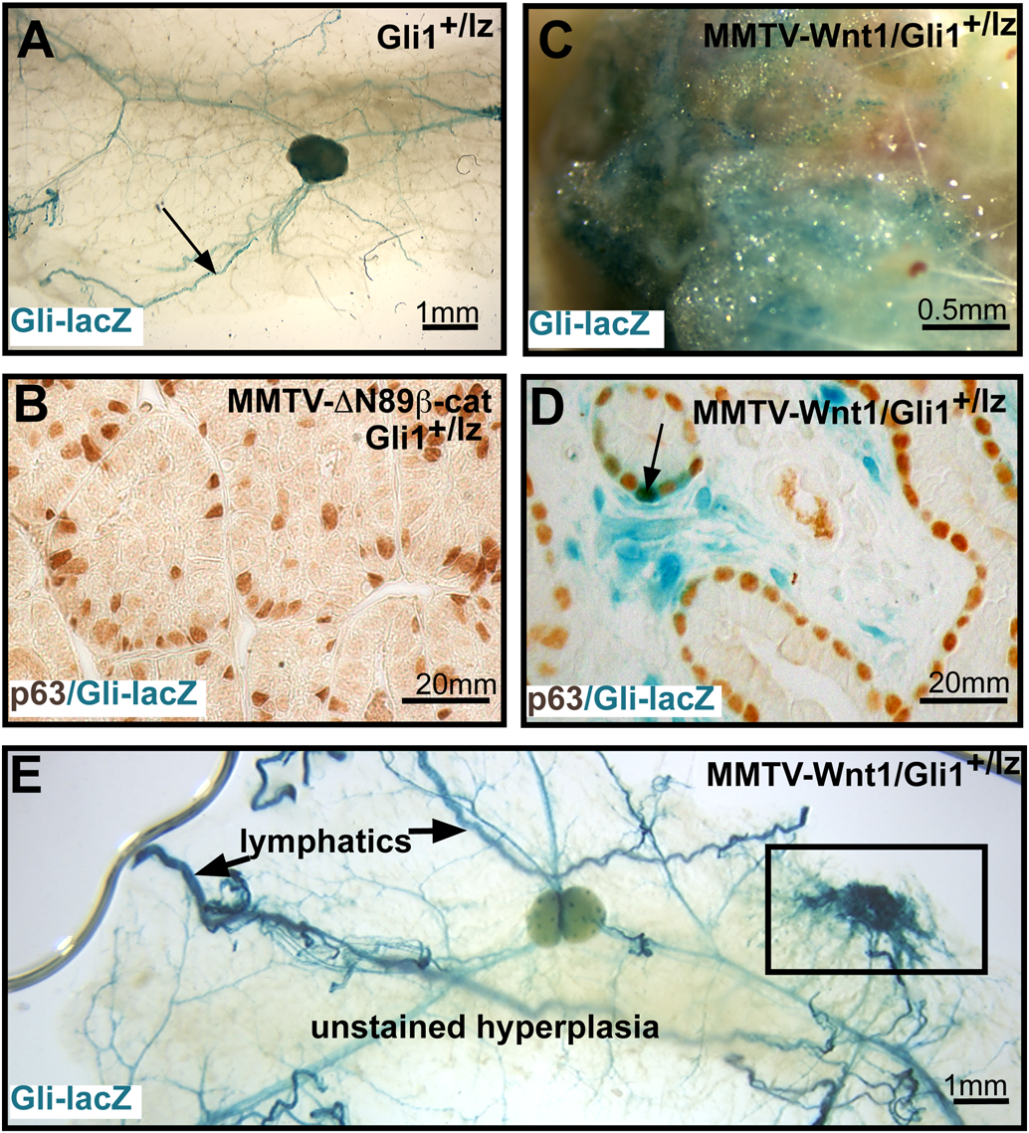
Hh pathway activity within the MMVT-Wnt1 tumor microenvironment. Gli1-lacZ expression is restricted to lymphatic vessels (arrow) in mammary whole mounts of (A) control Gli1^+/lz^ mice, and absent from tumor sections from (B) MMTV-ΔN89β-catenin;Gli1^+/lz^ mice, but is strongly expressed in (C) MMTV-Wnt1;Gli1^+/lz^ tumor whole mounts. In sections of MMTV-Wnt1;Gli1^+/lz^ tumors (D) Gli1-lacZ expression is found within stromal cells and occasional p63^+^ cells (arrow). (E) Hyperplastic MMTV-Wnt1;Gli1^+/lz^ mammary whole mounts from 12 week-old mice show lack of reporter expression within unstained mammary hyperplasia and restriction to lymphatics and melanocytic hyperplasia (boxed area).

Wnt and Hh pathways are frequently coupled in the cross-talk between stem cells and their stromal niche and an essential linkage between these pathways has been documented recently in basal cell skin cancer [35]. To determine if the Hh pathway was activated in hyperplasia and tumors of MMTV-Wnt1 and MMTV-ΔN89β-catenin mice, we crossed these lines to *Gli1^+/lz^* reporter mice. Hh signals are transduced by members of the Gli protein family, (Gli1-3). Gli1 is both a transcriptional target gene and positive amplifier of the pathway. Its expression is strictly dependent upon Hh signaling and thus provides a reliable reporter of Hh pathway activation [36]. Hh pathway activity is repressed within the mammary tree of non-transgenic *Gli1^+/lz^* control glands and *Gli1-lacZ* reporter expression was restricted to lymphatics (Fig. 6A) [37]. Similarly, hyperplasia and tumors from MMTV-ΔN89β-catenin;*Gli1^+/lz^* mice showed no evidence of *Gli1-lacZ* expression within the epithelial and stromal compartments (Fig. 6B). In contrast, tumors derived from MMTV-Wnt1;*Gli1^+/lz^* mice showed robust *Gli1-lacZ* expression within stromal cells and a minor subset of basal cell-types, some of which expressed p63 (Fig. 6C, D). Importantly, this reporter expression was restricted to the tumor bed and not observed in hyperplasia (Fig. 6E). These data demonstrate that Wnt1 expression leads to aberrant Hh pathway activation within the Wnt1 tumor microenvironment.

### MMTV-Wnt1 expression results in dramatic accumulation and differentiation of melanocytes along the lactiferous sinus and primary ducts

**Figure 7:**
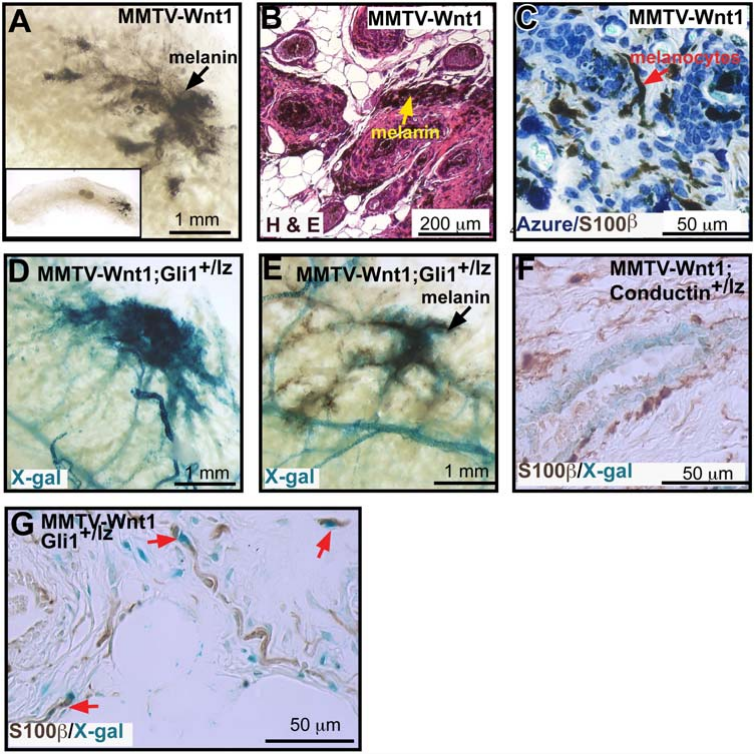
Melanocytic hyperplasia associated with Hh pathway activity is a hallmark of all MMTV-Wnt1 mammary glands. Mammary whole mount and sections from pigmented MMTV-Wnt1 mice show melanin deposition (A, B) and presence of melanocytes (C) detected by anti-S100β (DAKO #Z0311). Whole mounts and sections from albino (D, G) and pigmented (E) MMTV-Wnt1;Gli1^lz/+^ mice show Gli1-lacZ expression around the primary ducts localizing with melanin (E). Conductin-lacZ expression (blue) does not colocalize with S100β^+^ (brown) (F). However a minor subset of S100β^+^ melanocytes (brown) (red arrows) and many of their neighboring stromal cells express the Gli1-lacZ reporter (blue) (G).

Another striking linkage between Hh pathway activation and MMTV-Wnt1 expression was found in melanocytic hyperplasia under the nipple region (Fig. 6E box). A hallmark of mammary glands from pigmented MMTV-Wnt1 mice was accumulation of melanin around the trunk and primary mammary branches (Fig. 7A, E). Large black aggregates emanating from the nipple mesenchyme formed spider-like extensions into the mammary fat-pad that could be observed by eye (n = 29). These were absent from mammary glands of pigmented *wt* (n = 17) and MMTV-ΔN89β-catenin (n = 5) mice and from all albino mice regardless of genotype (n = 34 Wnt; n = 10 ΔN89β-catenin; n = 13 wild-type). Mammary whole-mounts (Fig. 7A) and sections (Fig. 7B–C) from pigmented MMTV-Wnt1 mice revealed melanin deposition within the basal layer, stromal cells and occasionally within the secretions of the primary lactiferous ducts. However, immunohistochemical analysis with antibodies against the S100β melanocyte marker revealed large numbers of dendritic melanocytes in albino as well as pigmented Wnt1 mice (Fig. 7C, G). We conclude that Wnt-1 expression leads to migration, accumulation and differentiation of melanocytes along the primary mammary ducts and that these cells are not present in MMTVΔN89β-catenin and *wt* mice.

To investigate whether Wnt1 induces these effects on melanocytes through canonical signaling and/or involves Hh pathway activation, we examined whole mounts of bitransgenic MMTV-Wnt1;*Conductin^+/lz^* and MMTV-Wnt1;Gli1*^+/lz^* mice for reporter expression. Conductin-Gal was restricted to basal cells and absent from the underlying stroma where melanocytes reside (Fig. 7F). In contrast, prominent *Gli1-lacZ* reporter expression was observed exclusively around the primary ducts of all MMTV-Wnt1;*Gli1^+/lz^* mice (n = 8) (Fig. 7D, E), colocalizing with the melanotic aggregates of pigmented MMTV-Wnt1;*Gli1^+/lz^* mice (n = 4) (Fig. 7E) and within a subset melanocytes identified by S100β staining and many neighboring fibroblasts in albino MMTV-Wnt1;*Gli1^+/lz^* mice (n = 4) (Fig. 7D, G). *Gli1-lacZ* expression was absent from corresponding regions of MMTV-ΔN89β-catenin;*Gli1^+/lz^* (n = 4) and control *Gli1^+/lz^* (n = 2) glands. We conclude that MMTV-Wnt1 expression induces aberrant melanocyte accumulation and differentiation within the fatpad and this event does not involve canonical pathways but correlates with robust Hh pathway activity within the stroma and a minor melanocyte subpopulation.

## Discussion

The key findings of this study are that MMTV-ΔN89β-catenin and MMTV-Wnt1 induce distinct tumors, activate canonical signaling in disparate cell-types, and MMTV-Wnt1 alone results in focal Hh pathway activity within the mammary tumor microenvironment and in and around melanocytic hyperplasia.

### MMTV-ΔN89β-catenin and MMTV-Wnt1 induce canonical signaling in distinct cell-types

Previous studies have shown that MMTV-ΔN89β-catenin and MMTV-Wnt1 tumors express primitive cell markers and are enriched in cell populations with stem/progenitor features [19]–[21], [34]. Our work extends these findings by identifying the cell-types that undergo a canonical response to transgene expression. Despite uniform MMTV-ΔN89β-catenin expression within the luminal epithelium [13], our results show that a specific subset of luminal cells express the transcriptional reporter, Conductin-*lacZ*. These β-catenin-responsive cells display a hormone-receptor-negative [38], K18^+^CD24^high^CD49f^low^ profile, and generate colonies containing both K8^+^ and K14^+^ cells. Thus, they exhibit the expected location, molecular profile and functional bipotency ascribed to alveolar progenitor populations from normal mammary glands [21], [27]. These features, in conjunction with the overwhelmingly alveolar-like phenotype of MMTV-ΔN89β-catenin hyperplasia, strongly support the hypothesis that MMTV-ΔN89β-catenin acinar tumors develop from expansion of alveolar progenitors, or by skewing the lineage of a bipotent alveolar/ductal progenitor towards an alveolar fate. This concept is reminiscent of K14-ΔN80β-catenin effects on skin appendage fate and consistent with our previous findings that known regulators of alveolar progenitor determination, expansion and differentiation influence the MMTV-ΔN89β-catenin-phenotype [18], [38].

Many studies have assumed that MMTV-ΔN89β-catenin and MMTV-Wnt1 exert a similar tumorigenic effect and have studied these models interchangeably. Where phenotypic differences have been noted Wnt1's potential to activate non-canonical pathways has been speculated upon but not experimentally addressed. Our results show that one definitive source of disparity between these models lies in the failure of Wnt1 to induce autocrine canonical signaling and its ability instead to induce a robust paracrine canonical response within a basally located K14^+^ cell population. Several pieces of data suggest that this Wnt1-responsive K14^+^ cell population consists of a stem cell or early bipotent progenitor population. First, the EGFP^hi^ expressing cells within the CD24^low^CD49^high^ subpopulation is skewed towards higher CD49f expression, a feature associated with MRU capacity, and depleted of cells with lower CD49f expression, a feature associated with mature myoepithelial populations [27]. EGFP^hi^ responding cells that segregate into the CD24^high^CD49^low^ group are also skewed rightwards and express K14, a profile recently ascribed to a CD61^+^ bipotent progenitor population shown to have cancer stem cell properties [22], [28]. Second, luminal and basal cell-types from MMTV-Wnt1 tumors share common genetic changes suggesting that the cell-type transformed by Wnt1 is a progenitor of both lineages [19]. Third, Wnt1 produces ductal defects (see fig 1) in addition to suppressing alveolar secretory differentiation [39]. Collectively, these results suggest that MMTV-Wnt1 induces expansion of a multipotent basal cell-type that precedes commitment to the ductal/alveolar as well as luminal/basal lineages.

### Wnt1 leads to Hh pathway activity in melanocytic hyperplasia and their surrounding stroma

A further source of disparity between the MMTV-ΔN89β-catenin and MMTV-Wnt1 phenotypes relates to the ability of the latter to affect additional surrounding cell-types. Of note, stromal hyperplasia (see Fig. 2L and 3E) is prominent within the early hyperplastic glands and tumors of MMTV-Wnt1 mice but is absent from glands and tumors of MMTV-ΔN89β-catenin mice. Another striking example is the dramatic effect on melanocyte populations. Our results show that massive melanotic deposits within the mammary fatpad are a hallmark of pigmented MMTV-Wnt1 mice and that large numbers of amelanotic melanocyte precursors accumulate around the primary lactiferous ducts of both pigmented and albino MMTV-Wnt1 animals. Murine melanocyte precursors normally reside in the hair follicle bulge and are not detected in the mammary fatpads of *wt* or MMTV-ΔN89β-catenin mice. Although both canonical and non-canonical Wnt signaling have been implicated in promoting melanocyte proliferation, migration and differentiation at other body sites [40], the lack of Conductin-*lacZ* reporter expression within the mammary melanocytic hyperplasia strongly favors non-canonical signaling. Intriguingly, our results show a tight correlation between positive Hh signaling within a minor melanocytic subpopulation and their surrounding stromal neighbors and the massive accumulation of mobilized, differentiating melanocytes in MMTV-Wnt1 glands. Current literature highlights a dualistic role for Hh signaling in melanocyte biology [41], [42]. Gli3R repression of Hh signaling is essential for melanoblast specification at certain body regions during development. However, consistent with our suggestion that Hh pathway activity is linked to melanocytic hyperplasia, one report has shown that positive Hh signaling is a critical determinant of melanoma growth and metastasis [42]. Importantly, our observations show that Wnt and Hh pathway activity are linked in the induction of these melanocytic accumulations and suggest that these pathways may contribute to pigmentary changes arising close to the nipple in association with an underlying breast carcinoma.

### Wnt1 leads to Hh pathway activity within the mammary tumor microenvironment

A second major site of focal Hh pathway activity is found within MMTV-Wnt1 tumors. Of note, Hh pathway activity was restricted to the Wnt-1 tumor microenvironment and was not observed within the Wnt-1 hyperplasia and thus correlated with Wnt1 tumor onset. Hh pathway repression is essential for embryonic mammary development and repression remains critical to homeostasis in the postnatal gland [37]. Pathway activation distorts mammary development, and is found in both epithelial and stromal compartments of human breast cancers [37], [43]–[46]. Intriguingly, *Gli1-lacZ* is expressed within a minor subset of p63^+^ basal cells in Wnt1 tumors and Ihh has been proposed to regulate proliferation of progenitors from stem cells via regulation of p63 isoform expression and to promote human mammosphere formation, considered to be a surrogate *in vitro* assay of stem cell proliferation [47], [48]. However the most prominent site of Hh pathway activity in MMTV-Wnt1 mice occurs in stromal cells exclusively within the tumor microenvironment. Whether stromal Hh pathway activation is essential for breast tumor onset remains to be determined. However, the importance of this possibility has been underscored by a recent study showing an essential role for stromal Hh pathway activity in supporting tumor cell growth of xenografted pancreatic and colonic human cancers and cell lines [49]. Given that MMTV-Wnt1 tumors are enriched in cells with stem/progenitor characteristics, we speculate that stromal Hh pathway activity may be indicative of the step when CSCs co-opt bystanders to become complicit in tumor formation by forming a supportive CSC niche. Currently the relative importance of Hh pathway activity within epithelial and stromal compartments of tumors is a matter of heated debate [49], [50]. As MMTV-Wnt1 tumors recapitulate the pattern of activity reported for human breast tumors they may provide as a useful genetic model to dissect the contribution of Hedgehog signaling within these different cell types to tumor onset and progression.

In summary our data show that Wnt1 and ΔN89β-catenin generate distinct tumors at least in part through activating canonical signaling in distinct progenitors and through Wnt-specific paracrine effects on multiple cell-types. Our data also show that Wnt 1 expression is linked with focal Hedgehog pathway activation in melanocytic nevi and mammary tumors. These data suggest Hh pathway activity and pigmentary changes may have utility as indicators of breast tumor onset induced by excess or unopposed Wnt ligand.

## Materials and Methods

### Ethics Statement

Animal maintenance and experimental procedures were in accordance with the NIH Guidelines for Animal Care and Use and were approved by the Institutional Animal Care and Use Committee of New York University Medical School.

### Mice

MMTV-Wnt1 and MMTV-ΔN89β-catenin mice were as described [13]. Axin2-d2EGFP, *Conductin^+/lz^*, Gli1*^+/lz^*, *Ltpb1L^+/lz^* were provided by Drs. Franke Costantini, Columbia University, Alexandra Joyner, Sloan Kettering Institute and Velocigene, Regeneron Pharmaceuticals, Inc. [32], [33], [36], [51].

### Mammary Whole Mount Staining

To detect *lacZ* expression, mammary glands and tumors were fixed in 4% paraformaldehyde (PFA) in 1× phosphate buffered saline (PBS) (PFA; Sigma Aldrich, St. Louis, MO) for 1 h, washed thrice in rinse buffer (2 mM MgCl_2_, 0.1% sodium deoxycholate, 0.2% NP40 in PBS) for 1 h each and stained overnight at room temperature in X-gal staining solution (50 mg/ml 5-bromo-4-chloro-3-indolyl-β-D-galactopyranoside in rinse buffer containing 5 mM potassium ferricyanide, 5 mM potassium ferrocyanide) (Applichem, Cheshire, CT). After staining, glands and tumors were rinsed in 1× PBS, post-fixed in 4% PFA overnight and processed for paraffin embedding. The tissues were sectioned, counterstained with Nuclear Fast Red (Polyscientific, Bay Shore, NY) and mounted for histological analyses. For whole mount analysis, X-gal stained mammary glands were counterstained with carmine, cleared and mounted as described http://mammary.nih.gov/tools/.

### Histology

Tissues were either fixed in PFA and X-gal stained as described above or fixed in 10% phosphate buffered formalin overnight, then processed and embedded in paraffin. 4 µ sections were deparaffinized with xylene (or citrosolve for X-gal stained tissue) and rehydrated through a graded series of ethanol. Citric acid antigen retrieval was performed for all antibodies by microwaving in 6.53 mM sodium citrate pH 6.0 for 30 min at 1.21 kilowatts. Rabbit antibodies against K14 (cat# PRB155P, Covance, Berkeley, CA) (1∶2000), MUC1 (1∶500) (Abcam), laminin (1∶100) (Sigma), S-100β (1∶4000)(Dako #Z0311), mouse antibodies against K18 (cat# 61028, Progen Biotechnik, Heidelberg, Germany), estrogen receptor (1∶500) (DAKO, Carpinteria, CA), and guinea-pig anti-vimentin (1∶1000)(Progen) were detected using biotin labeled secondary antibodies (Vector Labs, Burlingame, CA) (1∶1000) in conjunction with streptavidin peroxidase (Fisher Scientific, Suwanne, GA) that was colorimetrically detected using diaminobenzidine (cat# K3466, DakoCytomation, Carpinteria, CA). For S-100β staining, sections were subjected to antigen retrieval with 0.1% trypsin (Sigma #T0303), 0.1% CaCl2 in 50 mM Tris-HCl, pH 7.6 at 37°C for 45 min in a humidified chamber. Slides were washed continuously under running tap water for 5 min and processed for immunohistochemistry. Azure B staining was performed as described [52].

### Mammary Epithelial Cell Isolation and Flow Cytometry

Mammary glands and tumors were minced and digested for 6–7 h at 37°C in EpiCult-B Medium supplemented with 5% fetal bovine serum (FBS) containing 300 U ml^−1^ collagenase and 100 U ml^−1^ hyaluronidase. Digests were pipetted 8–12 times and vortexed for 5 seconds every hour. After dissociation, cells were pelleted at 350×g for 5 min and resuspended in a 1∶4 mixture of Hanks' Balanced Salt Solution containing 2% FBS (HF) and ammonium chloride and centrifuged again. Cell pellets were gently dissociated for 3 min in 5 ml of pre-warmed 0.25% trypsin-EDTA. 10 ml of cold HF was added. Cells were pelleted by centrifugation and resuspended for 1 min in 5 mg ml^−1^ of pre-warmed dispase II containing 0.1 mg ml^−1^ DNase I, then diluted with 10 ml of cold HF and filtered through a 40-µm mesh. All reagents were from StemCell Technologies Inc. [27]. All antibodies were purchased from BD Pharmingen unless otherwise noted. Cells at a concentration of 2.5×10^6^ per 100 µl of 2% bovine serum albumin (BSA) were incubated on ice for 30 min with primary antibodies: 0.5 ng/ml of biotinylated TER119 (cat# 553672), CD31 (cat# 558737), CD45 (553078), CD24-phycoerythrin (PE) (cat# 553262), and 0.25 ng/ml of CD49f-allophycocyanin (APC) (cat# FAB13501A, Minneapolis, MN, R&D Systems). Cells were washed once in 200 µl 2% BSA, stained with 1.5 ng/ml of streptavidin-Alexa488 on ice for 30 min, washed again and resuspended in 2% BSA. 1 µg/ml of 7-aminoactinomycin D (7-AAD) (cat# 00-6993-50, San Diego, CA, eBioscience) was added just prior to data acquisition. Flow cytometric data was acquired on a FACS Caliber cytometer at the NYU Skirball FACS Core Facility and analyzed using FlowJo software [27]

### Colony-Forming Assays

NIH-3T3 cells were irradiated in suspension at 20Gy and seeded as a feeder layer 24 h prior to assay, at a concentration of 1.9×10^4^ cells/well in 24-well plates (Corning Incorporated, Corning, NY). Lineage^+^ cells were depleted prior to sorting using the EasySep Cell Biotin Selection Kit (StemCell Technologies Inc.) using TER119, CD31, and CD45 and CD140A (eBioscience) antibodies. GFP^+^, GFP^−^ and Total Sorted tumor cells were sorted into 1 ml of 100% FBS using a MoFlo cell sorter, excluding cell debris, cell doublets, and autofluorescent cells. Sorted cell populations were centrifuged at 350 g for 3 mins, resuspended in a 300 µl, assessed for viability in 50% Trypan Blue and plated at limiting dilution. Media (EpiCultB+bullet, 5% FBS, 100 U/ml Penicillin/Streptomycin, 1 µg/ml Ciprofloxacin, 0.25 µg/ml Fungizone) was changed 48 h later to medium lacking FBS. Colonies were fixed after 6 days in 50∶50 acetone∶methanol at −20°C for 3 min and counted. Cells were stained with primary antibodies to K8 (Progen Cat#65138) and K14 (1∶2,000, Covance PRB-155P) for 30 min, washed three times in PBS, stained with goat anti-mouse (1∶100, Invitrogen Cat#A11801) and donkey anti-rabbit (1∶100, Chemicon Cat#AP182C) followed by three washes in PBS.
