# Correction: MMTV-Wnt1 and -ΔN89β-Catenin Induce Canonical Signaling in Distinct Progenitors and Differentially Activate Hedgehog Signaling within Mammary Tumors

**DOI:** 10.1371/annotation/3706d475-e082-4be6-b328-7d8aea02b986

**Published:** 2009-03-04

**Authors:** Brigitte Teissedre, Alicia Pinderhughes, Angela Incassati, Sarah J. Hatsell, Minoti Hiremath, Pamela Cowin

The following information was omitted from the funding statement: This work was also supported by NIH-R21-CA129905 and DOD-BCRP-W81XWH-08-1-0668. 

